# Health impact assessment and short-term medical missions: A methods study to evaluate quality of care

**DOI:** 10.1186/1472-6963-8-121

**Published:** 2008-06-02

**Authors:** Jesse Maki, Munirih Qualls, Benjamin White, Sharon Kleefield, Robert Crone

**Affiliations:** 1 Harvard Medical School, Harvard University, Boston, USA; 2 Harvard Medical International, Harvard University, Boston, USA

## Abstract

**Background:**

Short-term medical missions (STMMs) are a well-established means of providing health care to the developing world. Despite over 250 million dollars and thousands of volunteer hours dedicated to STMMs, there is a lack of standardized evaluation to assess patient safety, quality control, and mission impact. The objective of this project is to design and implement an assessment tool that defines objective parameters of quality of care as identified by STMMs.

**Methods:**

The study was conducted in 3 phases: 1) Base-need analysis to determine factors critical to the quality of STMMs, 2) Design of 5 surveys for mission personnel and patients to enable 360-degree evaluation based on factors from phase 1, and 3) Field testing of the surveys with 5 STMMs.

**Results:**

An evaluation tool was created assessing 6 major and 30 minor factors identified as important to the quality of STMMs. 5 mission directors, 43 personnel, 10 local hosts, and 55 patients completed the surveys. Of the 6 major measures of quality, missions performed best in Cost (mean score 86%), and Impact (84%). The poorest performance was in Education (64%). Efficiency, Sustainability, and Preparedness showed mean scores of 76%, 77%, and 73%, respectively.

**Conclusion:**

Our study provides a novel standardized tool for STMM evaluation. Use of the assessment instrument identified areas of strength and weakness of a particular mission, and delineated general trends in performance compared to other STMMs. We anticipate that the use of this tool may improve the quality of care provided by missions, and stimulate solution-sharing and scholarly discussion among missions.

## Background

In recent years, short-term medical missions (STMMs) have become a well-established vehicle for extending the reach of health care professionals to the developing world. They appeal to physicians and other medical professionals due to their unique combination of philanthropy and direct approach to patient care [1]. The National Library of Medicine MeSH heading defines "Medical Missions, Official" as "travel by a group of physicians to a foreign country for the purpose of making a special study or of undertaking a special study of a short-term duration [2]. This broad definition encompasses a wide range of services, ranging from surgical missions providing craniofacial reconstruction or cataract extraction [3,4] to medical and/or pediatric missions providing care for acute illness and chronic disease [5]. These differences extend to the organizational structure of STMMs. Missions differ widely in size (2 to 90 health care providers per mission) [6], budget (from a few hundred dollars to $39 million in annual expenses) [7], and duration (from 2 days to a month in length) [8]. Equally important, the emphasis on logistical detail is highly varied between missions. Paucity of follow-up data, poor relations with the local health care system, and lack of sustainability can challenge the good intentions of missions.

The number and popularity of STMMs have continued to rise, and considerable financial and human resources are expended on providing these services. While there is no official or complete compendium of medical missions, a search of the 3 largest mission websites – the International Healthcare Opportunities Clearinghouse [9], Diversion Magazine [10], and MissionFinder.org [11] – yielded a list of 543 medical mission organizations. Each of these organizations sends anywhere from 3 to 20 missions per year, for an annual total of approximately 6000 short-term missions sent to foreign countries from the United States. Some of these STMMs are large and well recognized, such as Mercy Ships, Project Hope, and Operation Smile, but the majority is sponsored by smaller groups and is known only to the people directly involved with the missions. Thus, with the 543 organizations listed sending an average of 10 missions per year at an average expenditure of $50,000 per mission, a very conservative estimate of annual expenditure on STMMs is $250 million but may be considerably more than that.

Despite this notable cost expenditure and the number and scope of STMMs, there is a paucity of literature on the subject. Without proper evaluation standards, issues of patient safety, quality control, and impact assessment are easily overlooked since STMMs are often locally organized and privately funded without restrictions. This can lead to disastrous results, such as 2 patient deaths after cleft-lip and palate repair, a result that would lead a malpractice suit in most Western countries [12]. Most STMMs have no objective means of measuring their performance and may lack formalized problem-solving techniques and methods for improvement. A MEDLINE [13] search between the years 1970 and 2006 for the terms "short term medical missions", "medical brigades", alone and in conjunction with the terms "safety", "quality", "impact" and "evaluation" returned a total of only 6 relevant results: 1 case report in English, 1 case report in Italian, 2 nursing articles, and 2 military articles. Thus, a means to evaluate STMMs is an important and needed step towards quality improvement in the realm of international health care.

The goal of this study was to create a systematic way to evaluate the performance of STMMs, and to use this tool to foster self-analysis and quality improvement within missions. We anticipate that this would be beneficial to both the providers and recipients of STMM care. A more critical approach to the delivery of care may lead to fewer wasted resources, higher patient satisfaction, and an all over improvement in the efficacy of medical care provided by STMMs.

## Methods

### Definition of Quality

The definition of quality offered by the Institute of Medicine was "the degree to which services for individuals and populations increase the likelihood of desired health outcomes and are consistent with current professional knowledge [14]." Additionally, care should be "safe, effective, patient-centered, timely, efficient, and equitable [15]."

### Design

Our study was conducted in 3 phases: 1) base-need analysis to determine factors relevant to the quality of STMMs, 2) surveys designed for mission personnel to self-evaluate their STMM, and 3) field testing of the surveys with 5 STMMs and subsequent response analysis. These phases are described below. The Harvard Medical School Institutional Review Board approved all procedures and protocols.

### Phase I – Base-need Analysis

Needs assessment was conducted with missions in Honduras, Guatemala and Venezuela. Selected missions had to meet the following criteria: 1) more than 5 years experience, 2) sponsor and direct at least 5 international missions per year, 3) allow one of the authors to participate in a mission and 4) commit to engaging in discussions with the author regarding factors pertinent to mission quality. Using MissionFinder [11], we investigated the online material for 40 missions; 28 were requested to participate in the study via email. Of the 13 missions that responded affirmatively, 6 were selected to represent a broad heterogeneity of size, medical goals and social affiliations to allow for greater generalizability of data. We conducted a total of 20 in-depth interviews with program directors, personnel, and recipients to answer the question: "What are the most important factors in evaluating the quality of STMMs." The interviews in this first phase of survey development were formatted with open-ended questions intended to facilitate discussion between the authors and mission personnel. The content of the interviews addressed the goals of STMMs, the logistics involved in providing health care in foreign country, and what benchmarks missions use to assess the quality of their work. Several factors were highlighted as relevant to the quality of STMMs during these interviews, but only 6 were discussed uniformly by all of the missions. These 6 points of commonality informed the 6 major factors that were incorporated into the surveys since they were benchmarks common to all missions regardless of mission type, service provided, and health care goal. Thirty other factors identified as impacting quality by at least 1 of the STMMs were designated as minor factors.

### Phase II: Survey Design

The 6 major factors and 30 minor factors identified during the base-needs analysis became the basis for survey questions. The 6 major factors were defined as:

#### Cost

A measure of the awareness of the total financial expenditure of the mission, and accuracy of assessing cost, including per patient cost, and the factors that influence it.

#### Efficiency

A measure of productivity; comparing measurable outcomes, such as the number of patients treated and complication rates, to time and resources spent.

#### Impact

A measure of the quality and effectiveness of the collective medical interventions as perceived by patients and providers.

#### Preparedness

A measure of the ability to function as an effective team, with other medical missions, and within the overall context of the medical system of the host country.

#### Education

A measure of the resources directed to providing responsible and accurate education, mentorship, and training to recipients and local health care workers.

#### Sustainability

A measure of the long-term focus of the mission, including fostering independence through building local capacity.

Because all of the minor factors informed more than 1 major factor, we created a matrix [see Additional file 1] to structure the relationship between major and minor factors. For example, the mission's ability to keep records can be used to evaluate all 6 of the major factors: calculating a mission Cost, measuring Efficiency or Impact, using past experience to better prepare for the future, improving on the Educational goals, or predicting Sustainability.

Five surveys were created in total for the host/local provider, mission director, patients, personnel and mission administrator [see Additional files 2, 3, 4, 5, 6]. The surveys each took less than 10 minutes to complete, with the exception of the mission director survey, which required approximately 45 minutes. The patient and host/local provider surveys were available in English and Spanish. Translators were available for other languages. Due to rates of illiteracy in the countries served, all patients that were surveyed were done so orally, in their native language, either directly or through a translator. All mission personnel and 10% of patients were surveyed. The rationale behind the 5 surveys was to provide a 360-degree evaluation incorporating the range of perspectives from people involved in the mission in a variety of capacities. Each survey was a combination of yes/no, Likert scale and free response questions. There were 170 total questions: 18 general information questions, 17 host, 29 personnel, 17 patient, and 81 mission director questions. Cost is evaluated by 7 questions, Efficiency 17, Impact 41, Preparedness 29, Education and Sustainability by 21 each. Additionally, 34 questions were categorized as 'General Information' and were used to obtain demographic data about the mission, such as type of mission and patient population served.

Each question was assigned a maximum value of 5 and each response was assigned a corresponding point value. For example, a Sustainability question asked on the mission director survey was: *It is easy to refer a patient to a local specialist or other mission for treatment or follow up*. The answers were scored as follows: Completely Agree (5 points), Somewhat Agree (4 points), Indifferent (3 points), Somewhat Disagree (2 points), and Completely Disagree (1 point). Yes/No questions received a value of 1 or 5, respectively, depending on the polarity of the question posed. There were 21 open-ended questions that were not assigned points and thus were not factored into the overall score but responses were available to mission personnel as part of the feedback report. The total points scored from the quantitative questions for each major factor were calculated and presented as a percentage of total possible points.

**Figure 1:**
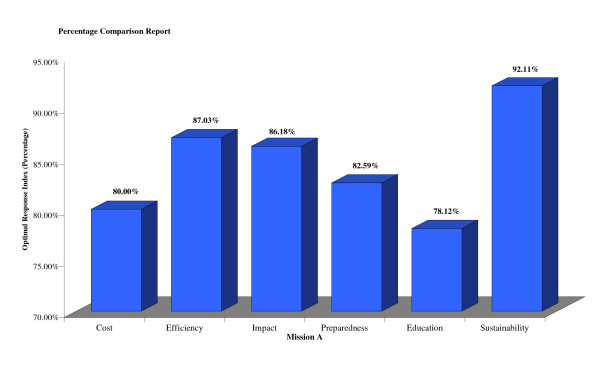
**Example Summary Report of 6 Major factors**. For a given mission, percentages are given representing scores for the 6 major factors. This allows the mission to view their scores for the 6 factors and compare the percentages within that mission to target quality improvement. In this example, the mission scored the highest in Sustainability and the lowest in Education. Thus, for this mission they may consider targeting their education initiatives as a means for quality improvement.

**Figure 2:**
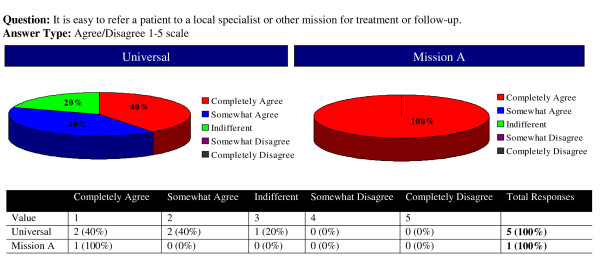
**Sample Survey Question on STMM Feedback Report**. This question is graphically represented with the Universal Average (the mean responses from all missions that have completed the surveys), compared alongside the specific answer from STMM A. In this sample question, the director of Mission A completely agreed that referral was easy compared to the universal average where 40% of missions completely agreed, 40% somewhat agreed and 20% were indifferent. The universal average of Likert response and standard deviation is also given in the graph as 1.8 and 0.7483 respectively.

The The The surveys were completed online or on paper and then entered into the online database: stmmconnect.org. A user account with password was provided to each mission, allowing, secure access to its own survey responses and to a feedback report that synthesized all the responses and represented them relative to other STMMs. The feedback report consisted of a percentile evaluation of the 6 major factors (Figure 1) allowing missions to rapidly and broadly analyze their performance. STMMs were also able to view responses to each individual question asked on the survey. While STMMs only had access to their own responses, they were able to compare their response on each Likert and yes/no question to the "universal average" which was the averaged score of responses of all other missions to each of these questions. (Figure 2) This feature again enabled a quick "snapshot" assessment of a particular STMMs performance on a more detailed level compared to other STMMs.

### Phase III: Pilot Testing

The final versions of the surveys were pilot tested with 5 STMMs in the catchment area of Honduras, Ecuador, Brazil, Zimbabwe, and Namibia. STMM organizations were selected based on the following criteria: 1) mission experience for more than 5 years, 2) direct at least 5 missions per year, 3) allow an author to survey the patients and personnel during a mission, and 4) provide written or verbal feedback regarding the surveys. The website and published material for approximately 75 missions was investigated, and 30 missions were queried regarding their interest in participating in using the survey-based evaluation tool. Of the 22 missions that responded, 5 were selected for maximum heterogeneity of size, locale, medical goals and social affiliations to allow for greater generalizability of data (Table 1).

**Table 1:** Mission Demographics

**Location**	**Type of care**	**Duration**	**Patients**
Brazil	Medicine	10 days	512
Honduras	Medicine/Pediatrics	6 days	350
Ecuador	Medicine	3 days	800
Zimbabwe	Surgery/Retina	4 days	50
Namibia	Surgery/Cataract	6 days	120

Demographics of the 5 missions surveyed including medical or surgical care provided, the durations of the missions, and the number of patients treated during the mission.

## Results

The needs assessment identified 6 major and 30 minor factors used to evaluate STMMs. These 36 factors were incorporated into 5 surveys. The surveys were piloted in 5 STMMs with results compiled from a total of 118 surveys – 5 general information, 10 host/local provider, 5 mission director, 43 personnel, and 55 patient surveys. The performance of each STMM was analyzed by the 6 major factors (Table 2).

**Table 2:** Parameter Scores by Mission

Mission	Brazil	Honduras	Ecuador	Zimbabwe	Namibia	Average
COST	100%	60%	90%	80%	100%	86%
EFFICIENCY	69%	67%	82%	87%	74%	76%
IMPACT	88%	87%	79%	86%	81%	84%
PREPAREDNESS	73%	65%	74%	83%	71%	73%
EDUCATION	75%	61%	51%	78%	53%	64%
SUSTAINABILITY	81%	67%	69%	92%	78%	77%

Overall scores for the 6 main major factors per mission. Scores are generated from self-reporting survey questions answered by the mission director, mission personnel, the local-host and patients.

Responses to Cost queries varied widely. The average per patient cost for medical missions was less than $3, while surgical missions averaged $700 per patient when accounting for surgical equipment used. Thus, total cost of medical and surgical missions ranged from $12,600 to $84,000 with an average of $34,400. No mission cited resource limitation as a barrier to care and 100% of missions stated that the total cost was actually less than or equal to the projected budget.

Impact was one of the highest scored major factors across missions. Fifty six percent of patients received medical treatment for the problem with which they presented. On average, patients waited for care between 30 minutes and 4 hours. Medical missions had between 1–3 days of follow-up care while surgical missions followed up at 1 day post-operatively and either day 3 or 1 week post-operatively. Twenty percent of mission personnel felt that the level of follow-up care was sufficient to accurately evaluate the impact of the mission. Seventy percent of missions reported 0–5% of patients treated returned with a complication of care delivered (wound infection, drug reaction). Thirty percent reported a 5–15% complication rate. The rate of mission reported satisfactory outcomes was 50–75% for 40% of missions, 75–90% for another 40% of missions, and greater than 90% satisfactory outcomes for 20% of missions. Over 75% of diagnoses were based solely on clinical presentation without the use of laboratory or radiological means to aid diagnosis.

In the Preparedness category 80% of missions reported an orientation session, 80% had a process for credentialing personnel, and 100% had an exit strategy in event of an emergency. However, 40% of missions did not have a system in place to collect morbidity and mortality data. Of missions that did collect morbidity and mortality data, 60% had a formal review of this information and linked with the local government for formal data reporting.

In terms of Sustainability, 80% of missions reported collaborating with the local health care system. Forty eight percent of patients report having another health care provider to turn to in case of an emergency or recurrence; 100% of missions report having a system in place to refer patients to another health care provider if needed. Patients had visited a given mission between 1 and 15 times, with an average of 3 recurrent visits.

The Efficiency parameter also identified areas in need of improvement. Sixty percent of missions reported triaging patients; 40% agreed that it is easy to refer patients to a local specialist, and 40% stated that there is an efficient communication system in place between team members.

The lowest average score across missions was in Education. It was often not a formalized goal of mission organizations. 60% of missions provided some training/education to the local or host doctors. Missions that did educate patients and providers used lectures, written media, and hands-on training of surgical techniques for local health care providers.

## Discussion

This tool represents an innovative approach towards evaluating a branch of international health services which have not yet adopted standardized quality measures. To our knowledge, this survey-based evaluation tool is the first evaluation tool of its kind for STMMs. Its content is based directly on research and feedback from mission professionals, as the cornerstone of developing an accurate, targeted assessment is the precise definition of quality as it pertains to a particular field [16,17]. The surveys address areas of concern common to most STMMs regardless of the specific goals of the mission organization. It investigates these common concerns via a 360-degree assessment through the responses of several informants: the director, host, personnel, and patient. This 360-degree evaluation approach is widely recognized as a quality improvement method which is able to assess multiple aspects of competence [18]. Integrating responses from the surveys allows the mission organization to assess the overall effectiveness of each individual mission and target areas for improvement – specifically in the 6 major and 30 minor factors evaluated in the surveys. By representing the 6 major factors as percentages and allowing for comparison of performance in each parameter, these surveys give STMMs a means to evaluate the quality of their mission, using their own goals as well as relative performance compared to other STMMs. Collaboration with other missions using these delineated factors is an important way to share strategies and solutions. This is further facilitated via the online database and website.

The surveys herein developed provide the first objective measure of STMM quality that can be standardized across missions. A single standardized core measurement set is the only way to identify differences between providers of care in such a way that good practices can be reliably differentiated from faulty practices [19]. Considerable effort has gone into focusing the development of these surveys on the needs of the missions and maintaining a user-friendly format. Missions have access to 5 surveys that can be completed quickly and easily, without interfering in the daily work of the mission. The feedback that is generated through the automated report offers important insight into the overall quality of the mission as well as areas for improvement.

In its current form, there are limitations to this evaluation tool. First, no authoritative international body currently oversees STMMs. Thus, our tool relies on medical mission directors to honestly evaluate their own missions. This is a source of potential bias towards positive evaluations, since mission directors are often invested both professionally and personally in the success of their missions. Secondly, many mission participants are intimately connected to their mission groups, either through religious affiliations, schools or places of employment. This sense of camaraderie may make it difficult for them to objectively evaluate the mission. Furthermore, many mission participants work in short-term medical missions for altruistic reasons. These good intentions directed towards disadvantaged populations can lead to the misconception that in resource-poor environments any healthcare is good healthcare, regardless of the quality of services. This misperception could also contribute towards a biased positive evaluation.

A final source of bias lies in the issue of interviewing patients. In the real-world setting of a medical mission, it is quite difficult to ensure that all patients complete the survey in a private, confidential environment. We have plans to translate the survey into several languages beyond its current English and Spanish versions. However, since many patients may be illiterate, the survey would ideally be a one-on-one interview with an impartial person who is fluent in the local language. Missions should decide ahead of time what percentage of patients they wish to interview, and then randomly select patients as they leave the clinic, i.e. sampling every third patient for a thirty percent sample size. This requires at least one full-time staff person to be dedicated towards eliciting patient feedback. In our experience, there are usually several members of medical missions who do not have medical training; this would be an ideal role for those participants.

These potential biases impact the interpretation of the percentages by the missions. From our results, it is clear that the percentages in all areas were relatively high, all greater than 60%, which may be a product of self-evaluation. We emphasize that the percentages are not meant to be read as stand-alone values, but rather as a relative comparison to the percentages scored by that mission on the other major factors and to the averaged score from other missions. The percentage scores can thus be used to identify target areas for improvement by objective means and to guide the mission on quality improvement using feedback generated by their own patients and personnel. We believe that introducing this tool is an important first step in a field that currently lacks any real quality indicators, and that this self-evaluation method is the most effective approach to change, given the current emphasis on leadership as an essential ingredient in the enterprise of quality improvement [20,21]. While this tool cannot provide a completely objective assessment of a mission, its application will help mission organizations and directors to think carefully about the services they provide. We hope that this process will facilitate their entry into the dialogue about quality that has been so important in other areas of healthcare.

This tool offers these missions a first step towards evaluating their performance from the perspectives of all involved parties. However, there is a great deal of variation between missions, both in type of care provided and in the operational details. In the future, we plan to look more critically at certain mission-specific outcomes (e.g. surgical vs. medical outcomes) and will augment the surveys with questions that specifically evaluate these different services. We will also provide questions on complications specific to certain types of missions so that mission organizations can refine their surveys to reflect their particular health interventions. Additionally, we plan on adding questions regarding patient selection, the level of education of those providing care, as well as how the care provided compares clinically with the care that would normally be provided in the host country as well as the country of the mission organization.

The method of data entry may also merit improvement. In most locations internet access is unreliable or non-existent, necessitating the delivery and collection of paper surveys followed by online data entry upon return to the United States or another location with internet access. This essentially doubles the time needed to complete the surveys. Current options to address this include having a central administrator for STMMConnect who receives the surveys in paper form at the end of the mission and then is responsible for the data entry. Alternatively, an administrator of the mission or the mission director can be asked to take responsibility for the surveys. In the future, it may be possible to send missions with a computer-based program that records the survey data and can then be linked to the internet whenever the mission director is able to attain online access.

Finally, financial forms are an important and needed part of this tool. Currently, the Cost parameter analyzes mission's awareness and perception of its total and per patient cost. It may be informative to also objectively assess mission costs including airline and lodging costs, resource utilization, as well as forfeited wages on the parts of the mission personnel for the participation in the mission.

## Conclusion

STMMs are an important component of global healthcare and a rapidly growing sector that accounts for millions of dollars of public and private funds. To date, no standardized assessment tool has been developed to assess the quality of STMMs. Here we provide an efficient and user-friendly tool for 360-degree self-evaluation focusing on quality and health impact assessment. We anticipate that application of this tool will stimulate scholarly discussion of common problems shared by STMMs and provide a venue for solution sharing through the online database: STMMconnect.org.

The literature on quality improvement and health impact assessment has called the delivery of quality, cost-effective care a "public good" [19,22]. Thus, a properly constructed, empirical evaluation method of health care delivery is essential. Here we introduce the first such evaluation tool for STMMs. We assert that STMMs are not exempted from this evaluation due to their altruistic and transient nature. Rather, given the high vulnerability and substantial medical needs of the populations they serve, STMMs in particular stand to benefit from a means to objectively inform the health care decisions they make. Looking more critically at mission specific outcomes can foster a discussion on how to optimize quality, address deficiencies, and solution-share in order to improve the quality of care offered to patients in developing areas worldwide.

## Abbreviations

STMM: short-term medical missions.

## Competing interests

The authors declare that they have no competing interests.

## Authors' contributions

BW carried out the initial surveys of missions and participated in the survey design. JM and MQ participated in survey design, and carried out the 2^nd ^phase of the study by testing the created surveys with the 5 missions reported in this study; both authors drafted this manuscript. SK was involved in the analysis of data and application of quality principles to the surveys. BC has been the chief advisor for this study and participated in the design of the surveys. All authors read and approved the final manuscript.

## Pre-publication history

The pre-publication history for this paper can be accessed here:



## Supplementary Material

Additional file 1 The matrix used which shows the Major and Minor factors used for evaluation and that were incorporated into the surveys. Survey used for the missions to self-evaluate. Click here for file

Additional file 2 Host/local provider survey. Survey used for the missions to self-evaluate. Click here for file

Additional file 3 Mission Director Survey. Survey used for the missions to self-evaluate. Click here for file

Additional file 4 Patient Survey. Survey used for the missions to self-evaluate. Click here for file

Additional file 5 Personnel Survey. Survey used for the missions to self-evaluate. Click here for file

Additional file 6 Administrative/General Information Survey. Survey used for the missions to self-evaluate. Click here for file
