# Supplementary material for: Health impact assessment and short-term medical missions: A methods study to evaluate quality of care
Source: BMC Health Serv Res. 2008 Jun 2;8:121. doi: 10.1186/1472-6963-8-121 (PMC2464597; doi:10.1186/1472-6963-8-121)
Supplement: Additional file 1 — The matrix used which shows the Major and Minor factors used for evaluation and that were incorporated into the surveys. Survey used for the missions to self-evaluate. [file 1472-6963-8-121-S1.doc]

### Additional file 1: STMM Survey Matrix

|  | **COST** | **EFFICIENCY** | **IMPACT** | **PREPAREDNESS** | **EDUCATION** | **SUSTAINABILITY** |
| --- | --- | --- | --- | --- | --- | --- |
| **Total Cost** | A1 | **-** | **-** | **-** | **-** | F1 |
| **Per Patient Cost** | A2 | - | - | - | - | F2 |
| **Host Cost** | A3 | - | - | - | - | F3 |
| **Financial Evaluation** | A4 | - | - | - | - | F4 |
| **Diagnostic Methods** | - | B5 | C5 | - | - | - |
| **Standard of Care** | A6 | B6 | C6 | D6 | E6 | - |
| **Measurable Outcomes Methods** | - | B7 | C7 | E7 | - | F7 |
| **Outcomes** | - | - | C8 | - | - | F8 |
| **Morbidity and Mortality** | - | B9 | C9 | - | - | F9 |
| **Staffing Plan** | A10 | B10 | - | D10 | - | - |
| **Patient Census** | - | B11 | C11 | D11 | - | - |
| **Services** | - | B12 | C12 | - | - | - |
| **Resources** | - | - | C13 | D13 | E13 | - |
| **Orientation** | - | - | - | D14 | E14 | - |
| **Risk Management** | A15 | - | C15 | D15 | - | F15 |
| **Language Proficiency** | A16 | B16 | C16 | D16 | E16 | - |
| **Cultural Awareness** | - | - | C17 | D17 | - | - |
| **Organization and Management** | A18 | B18 | C18 | D18 | - | - |
| **Triage** | - | B19 | C19 | - | - | - |
| **Referral Process** | - | B20 | C20 | D20 | - | - |
| **Time Management** | A21 | B21 | C21 | - | E21 | - |
| **Communication** | - | B22 | - | D22 | - | - |
| **Patient Health Education** | - | - | C23 | D23 | E23 | F25 |
| **Host provider Training** | - | - | C24 | D24 | E24 | F26 |
| **Resident/Student Training** | - | - | C25 | D25 | E25 | - |
| **Record Keeping** | A26 | B26 | C26 | D26 | E26 | F26 |
| **Exit Strategy** | - | - | - | D27 | - | F27 |
| **Clarity of Goals** | - | - | - | D28 | - | F28 |
| **Personnel Satisfaction** | - | - | - | - | - | F29 |
| **Patient Satisfaction** | - | - | - | - | - | F30 |

Each of the 6 major factors are evaluated by multiple minor factors that will allow a separate score to be calculated. Because a given minor factor can potentially be used as a surrogate measure for multiple major factors, a matrix was created to allow for stratification and scoring of each individual factors (major and minor). The 6 major factors are listed across the horizontal axis, with the minor factors on the vertical axis. Each intersection on the matrix with an alphanumerical code corresponds to a question or set of questions that is asked on a survey; those marked with a dash have no corresponding questions for that intersection of a given major and minor factor.
