# Supplementary material for: Health impact assessment and short-term medical missions: A methods study to evaluate quality of care
Source: BMC Health Serv Res. 2008 Jun 2;8:121. doi: 10.1186/1472-6963-8-121 (PMC2464597; doi:10.1186/1472-6963-8-121)
Supplement: Additional file 2 — Host/local provider survey. Survey used for the missions to self-evaluate. [file 1472-6963-8-121-S2.doc]

## Additional file 2: Host/Local Provider Survey

1. Please indicate the type(s) of care this mission provided.

Surgical Dental Medical Pediatric

1. Did local health care providers collaborate with the mission doctors?

YES NO

1. Was training provided?

YES NO

If training for local medical professionals was provided by the mission doctors, please describe your degree of satisfaction with it in questions 4 and 5:

1. The mission doctors taught local doctors useful new skills

#### Completely Agree---------------Completely Disagree

#### 1 2 3 4 5

1. The care local doctors provide as a result of this training is better than before

Completely Agree---------------Completely Disagree

1 2 3 4 5

1. There is good host-mission communication.

Completely Agree---------------Completely Disagree

1 2 3 4 5

1. The host and local health care providers were well integrated into the mission team

Completely Agree---------------Completely Disagree

1 2 3 4 5

1. Did you have any complaints about the organization (communication, travel arrangements, etc) of the mission?

YES NO (if YES please explain)

1. On average, how many days of follow-up care are provided to each patient?

__________________days

1. Is data collected about the clinical outcomes of patients?

YES NO

1. Do you feel that the data collected is sufficient to evaluate the care provided by the mission?

YES NO

1. What percentage of patients returned to local health care facilities with a problem or complaint that was due to the actions of the mission (adverse drug reaction, surgical wound infection, etc.)?

0-5% 5-15% 15-25% 25-50% 50-100%

1. Given the cost faced by the host for this mission, for how many years will the mission be sustainable at this site?

>1 year >5 years >10 years

1. Would you like the mission to return for another visit?

YES NO

1. Your overall experience with this mission was positive.

Completely Agree---------------Completely Disagree

1 2 3 4 5
