# Supplementary material for: Health impact assessment and short-term medical missions: A methods study to evaluate quality of care
Source: BMC Health Serv Res. 2008 Jun 2;8:121. doi: 10.1186/1472-6963-8-121 (PMC2464597; doi:10.1186/1472-6963-8-121)
Supplement: Additional file 3 — Mission Director Survey. Survey used for the missions to self-evaluate. [file 1472-6963-8-121-S3.doc]

#### Additional file 3: Mission Director Survey

1. How long have you been with this mission?
2. Besides director, what other roles do you play in the mission?
3. Please circle all applicable responsibilities in your role as director:
   1. financial information
   2. logistical organization on-site
   3. recruitment of participants
   4. assigning jobs to participants
   5. publicity/media
   6. fundraising
   7. communication with host country
   8. communication with mission team
   9. acquiring medical supplies
   10. medical care
   11. interacting with host country government
   12. other:
4. What are the primary methods of diagnosis used by your mission?

Circle all that apply

- 1. Chief Complaint
  2. History of Present Illness
  3. Past Medical History
  4. Family History
  5. Social History
  6. Physical Exam
  7. Laboratory Tests
  8. Radiology Tests (XR, CT, MR)
  9. Local Epidemiology
  10. Other

1. What is the average length of the diagnostic portion of the visit?

<5min 5-10min 10-15min >15min

1. What percentage of diagnoses were based solely on the clinical presentation of the patient?

0-15% 15-25% 25-50% 50-75% 75-100%

1. The accuracy of your diagnoses would be improved with access to laboratory and other diagnostic testing methods?

Completely Disagree---------------Completely Agree

1 2 3 4 5

1. Have you achieved your expected standard of care?

YES NO

1. If not, please identify which of the following were limiting factors? Circle all that apply.
   1. Cost
   2. Efficiency
   3. Personnel
   4. Pre-mission Preparation
   5. Cultural Competence
   6. Infrastructure
   7. Supplies/Medical Equipment
   8. Access to appropriate facilities
   9. other:____________________
2. Is there a system in place for measuring health outcomes in the patient population?

YES NO

1. The level of follow-up care is sufficient to accurately evaluate the impact of this mission on its patients.

Completely Disagree---------------Completely Agree

1 2 3 4 5

1. Are outcomes measured in such a way that they can be compared to the US Standard of care?

YES NO

1. What percentage of outcomes are satisfactory or better?

0-25% 25-50% 50-75% 75-90% 90-100%

1. On average, how many days of follow-up care are provided to each patient?.

__________________days

1. Is the level of follow-up data sufficient to accurately evaluate the impact of the mission?

YES NO

1. Does the mission routinely review outcomes and use them to inform subsequent missions?

YES NO

1. What percentage of patients returned to clinic with a problem or complaint that was due to the actions of the mission (adverse drug reaction, surgical wound infection, etc.) _________________%
2. Were there any fatal negative patient outcomes?

YES NO

1. Is there a system in place to collect morbidity and mortality data? Please describe briefly.

YES NO

1. Is there a system in place for review of morbidity and mortality data?

YES NO

1. Has the morbidity and the mortality data improved from year to year?

YES NO

1. How many patients were cared for over the course of the mission? ________________ patients
2. If this mission has been to this location before, has the number of patients cared for per mission increased or decreased from year to year?

Increased Stable Decreased

1. Which of the following limited the total number of patients treated? Circle all that apply.

1. Cost 2. Personnel 3. Time

4. Efficiency 5. Preparedness 6. Self-imposed

7. Supplies 8. other:___________

1. Is a formal staffing plan used to determine team membership?

YES NO

1. Did the mission have all the necessary personnel?

YES NO

1. What percentage of participants are essential personnel? Eg. Health care providers, translators, etc.

0-25% 25-50% 50-75% 75-90% 90-100%

1. Does the mission have a process for credentialing the participants?

YES NO

1. How many separate services does your mission provide? (A service is defined as a branch of medical or dental specialty). Please list:
2. Is there a system in place to track medical supplies used?

YES NO

1. Are any specific resources chronically in short supply? Please list below.

YES NO

1. Care was negatively affected by resource limitation (eg. lack of medical supplies, clinical spaces, etc. ).

Completely Disagree---------------Completely Agree

1 2 3 4 5

1. Does an orientation session take place prior to each mission? Please provide a copy of your orientation agenda.

YES NO

1. Are issues of cultural awareness integrated into the orientation session?

YES NO

1. Did any adverse events such as accidents or injury to participants take place during the mission?

YES NO

1. Were you well prepared to handle those events? If no, why not?

YES NO

1. Does the mission have a safety strategy plan in place to act quickly and efficiently in case of emergency.

YES NO

1. Is there a system in place for measuring the language proficiency of the team in the native language of the recipient patients?

YES NO

1. What percentage of patient and local provider education is performed in the native language?

0-15% 15-25% 25-50% 50-75% 75-100%

1. Is the host country’s ministry of health aware of your mission? If not, please explain.

YES NO

1. Did you receive approval from the country’s licensing board to bring these providers in country?

YES NO

1. How was the mission publicized?

word of mouth sign/flier radio health provider other:____________

1. Do you triage patients?

YES NO

1. If not, how do you determine which patients receive care?
2. Is there a system in place for referring patients to a local specialist or other mission if need be?

YES NO

1. If no, have any steps been taken to set up a referral network?

YES NO

1. It is easy to refer a patient to a local specialist or other mission for treatment or follow-up.

Completely Disagree------------------Completely Agree

1 2 3 4 5

1. Were any patients referred?

YES NO

1. Please categorize how participants spend their time during the mission in terms of the following activities (should add up to 100%)
   1. Patient Care __________%
   2. Patient Health Education __________%
   3. Education of Local Health Care Providers __________%
   4. Administrative/Logistical Duties __________%
   5. Team Building (social hours, discussions, etc.) __________%
   6. Religious activities in the community __________%
   7. “Down time” (rest, sleep, tourism, etc.) __________%
2. What is the average amount of time that patients wait for care?

__________________hours

1. There is an efficient communication system in place between team members.

Completely Disagree------------------Completely Agree

1 2 3 4 5

1. There is an efficient communication system in place between mission participants and local host.

Completely Disagree------------------Completely Agree

1 2 3 4 5

1. Is there a system in place for communicating with other missions in the same medical field or geographical area of practice?

YES NO

1. Were there any complaints about the logistical organization of the mission from the participants or host?

YES NO

1. Is there a system in place for providing patient health education while the patients are not receiving direct health care?

YES NO

1. Does a curriculum exist?

YES NO

1. Were the materials available for patient health education sufficient to achieve educational goals, if that is part of your mission statement?

YES NO

1. Education provided by the mission impacted the health awareness of the patient population.

Completely Disagree------------------Completely Agree

1 2 3 4 5

1. Is there a system in place for training local health care providers?

YES NO

1. Do opportunities exist for local providers to train mission providers?

YES NO

1. Does a curriculum exist for either training opportunity?

YES NO

1. Were the materials available for training local health care providers sufficient?

YES NO

1. Education provided by the mission positively impacted the level of care delivered by local health care providers.

Completely Disagree------------------Completely Agree

1 2 3 4 5

1. Is sustainable improvement and eventual independence of the host a goal of the mission’s effort at training local health care providers?

YES NO

1. Is it one of the goals of the mission to train residents and students?

YES NO

1. Does a curriculum exist?

YES NO

1. In terms of providing care to patients, are residents and students held to the same standards as in their country of origin?

YES NO

1. What is the total cost faced by your organization for this mission?

__________________USD (specify if other currency)

1. Based solely on the current financial situation of your organization and the cost of this mission, approximately how many years will the mission be sustainable?

>1 year >5 years >10 years

1. What is your average cost per patient?

__________________USD (specify if other currency)

1. What measures were taken to reduce per patient cost? Please list
2. Does an expected budget exist for each mission?

YES NO

1. Was the actual cost for the mission less than or equal to the expected budget calculated prior to the mission?

YES NO

1. It was easy to accurately answer the previous six questions.

Completely Disagree---------------Completely Agree

1 2 3 4 5

1. How many questions on the Mission Finances Form were you unable to complete due to lack of records?

1-2 3-5 6-8 9-10 >10

1. Does the mission keep records on every patient treated by the mission?

YES NO

1. Are they easily accessible?

YES NO

1. Can they be transferred to the patient’s home health care provider?

YES NO

1. Is the outcome of each treatment contained in his/her records?

YES NO

1. Are records from previous missions available to be used in preparation for future missions?

YES NO

1. Is there a system in place for keeping accurate records of the educational efforts of the mission?

YES NO

1. If building a sustainable healthcare system in country is a goal of this mission, does an exit strategy exist for the mission?

YES NO NA

1. Have factors been identified to determine implementation of the exit strategy?

YES NO

1. Has a “mission statement”, or some other statement of purpose or goals, been composed for the mission?

YES NO

1. Have criteria been established for judging the success of the mission?

YES NO

1. Do you ask your patients and participants for suggestions for improvement?

YES NO

1. Do you have a method for evaluating and integrating these suggestions?

YES NO
