# Supplementary material for: Health impact assessment and short-term medical missions: A methods study to evaluate quality of care
Source: BMC Health Serv Res. 2008 Jun 2;8:121. doi: 10.1186/1472-6963-8-121 (PMC2464597; doi:10.1186/1472-6963-8-121)
Supplement: Additional file 4 — Patient Survey. Survey used for the missions to self-evaluate. [file 1472-6963-8-121-S4.doc]

###### Additional file 4: Patient Survey

1. What medical problem are you dealing with today?
2. Did you receive medical treatment for this problem?
3. How did you get here today?

On foot public transportation personal vehicle other:________

1. How many times have you visited missions from this organization?
2. How often do you see a physician?

less than 1/yr 1-2/yr 6/yr monthly

1. Please rate your overall experience with this mission.

Poor Average Excellent

1. Approximately how long did you wait for care?

­ __________________hours

1. How well did you understand what the doctors/dentists/translators told you?

Poor Average Excellent

1. What did this mission teach you about your health?
2. Do you understand how to care for this problem in the future?
3. Will you be able to follow the advice of the doctors?

YES NO

1. Do you have access to another health care provider in case of a complication?
2. How did you hear about this mission?

word of mouth sign/flier radio health provider

1. Were you required to pay anything? If so, how much? Will this affect your living conditions in any way? Please explain.
2. Do you have any suggestions for improving this mission?
