# Supplementary material for: Health impact assessment and short-term medical missions: A methods study to evaluate quality of care
Source: BMC Health Serv Res. 2008 Jun 2;8:121. doi: 10.1186/1472-6963-8-121 (PMC2464597; doi:10.1186/1472-6963-8-121)
Supplement: Additional file 5 — Personnel Survey. Survey used for the missions to self-evaluate. [file 1472-6963-8-121-S5.doc]

#### Additional file 5: Personnel Survey

1. What is your affiliation with this mission? What type of service did you provide? (doctor, surgical nurse, public health educator, etc)
2. How many short-term medical missions have you participated in?

__________________Missions

1. How many missions have you participated in with this organization?

__________________Missions

1. The orientation meeting and orientation efforts of the mission were well organized and of high quality.

Completely Disagree---------------Completely Agree

1 2 3 4 5

1. Were there any complaints about the logistical organization of the mission?

YES NO (If yes, please list below)

1. Did the mission have all the necessary personnel?

YES NO (If no, please explain below)

1. Care was negatively affected by resource limitation (eg. lack of medical supplies, clinical spaces, etc.).

Completely Disagree---------------Completely Agree

1 2 3 4 5

1. The communications system in place for intra-team communication is efficient.

Completely Disagree---------------Completely Agree

1 2 3 4 5

1. The communications system in place for intra-team communication is beneficial to the mission.

Completely Disagree---------------Completely Agree

1 2 3 4 5

1. Participants’ time was spent efficiently during the mission?

Completely Disagree---------------Completely Agree

1 2 3 4 5

1. Please indicate what percentage of your time was spent on the following activities during the mission. This should add up to 100%.
   1. Patient Care __________%
   2. Patient Health Education __________%
   3. Education of Local Health Care Providers __________%
   4. Administrative/Logistical Duties __________%
   5. Team Building (social hours, discussions, etc.) __________%
   6. Religious activities in the community __________%
   7. “Down time” (rest, sleep, tourism, etc.) __________%

1. What percentage of diagnoses were based solely on the clinical presentation of the patient as opposed to lab data or medical records?

0-15% 15-25% 25-50% 50-75% 75-100%

1. On average, how long was the diagnostic portion of the patient visit?

<5min 5-10min 10-15min >15min

1. On average, how many days of follow-up care did you provide to each patient?

__________________Days

1. Is there a method to track the post-care health outcomes of your patient after s/he leaves the mission?

YES NO

1. Do you believe the level of follow-up care is sufficient to accurately evaluate the impact of this mission on its patients?

YES NO

1. What percentage of patients returned to clinic with a problem or complaint that was due to the actions of the mission? (adverse drug reaction, surgical wound infection, etc.)

0-5% 5-15% 15-25% 25-50% 50-100%

1. It is easy to refer a patient to a local specialist or other mission for treatment or follow-up.

Completely Disagree------------------Completely Agree

1 2 3 4 5

1. The interpreter services available during the mission are adequate to maintain a high level of care.

Completely Disagree------------------Completely Agree

1 2 3 4 5

1. Please rate your ability to speak the native language of the patients. Please circle.
   1. Basic (greetings, salutations, moderate understanding)
   2. Conversational (basic phrases and tenses, good understanding)
   3. Proficient (expert, idiomatic phrases, excellent understanding)
   4. Fluent (effortless expression, complete understanding)
2. Greater language proficiency would have increased your personal productivity.

Completely Disagree------------------Completely Agree

1 2 3 4 5

1. Please rate your knowledge of local culture before this mission. Please circle.
   1. I knew nothing about the culture.
   2. I knew a little about the culture.
   3. I was average in my knowledge.
   4. I was very comfortable with my knowledge.
   5. I know the culture as well as if it were my own.
2. Greater cultural awareness would have improved the quality of care provided.

Completely Disagree------------------Completely Agree

1 2 3 4 5

1. If teaching is part of your mission’s goals, the educational resources available to you were satisfactory.

Completely Disagree------------------Completely Agree

1 2 3 4 5

1. If you are a resident or student, the educational experience provided by the mission positively impacted the level of care you delivered or will deliver. Please comment if it impacted how you think of your future career or patient-doctor relationship.

Completely Disagree------------------Completely Agree

1 2 3 4 5

1. Your overall experience with this mission was positive.

Completely Disagree------------------Completely Agree

1 2 3 4 5
