# Supplementary material for: Health impact assessment and short-term medical missions: A methods study to evaluate quality of care
Source: BMC Health Serv Res. 2008 Jun 2;8:121. doi: 10.1186/1472-6963-8-121 (PMC2464597; doi:10.1186/1472-6963-8-121)
Supplement: Additional file 6 — Administrative/General Information Survey. Survey used for the missions to self-evaluate. [file 1472-6963-8-121-S6.doc]

**Additional file 6: Mission Administrator (General Information) Survey**

1. What is the name of the mission?
2. What location did this mission serve?
3. What were the dates of the mission?
4. Please list the mission director’s contact information here:
5. What services are provided by your mission? (Please choose all that apply)

Medical Surgical Dental Social

1. Do you have a mission statement? If so, please attach it to this document.

YES NO (If yes, please attach)

1. Does the mission have any affiliations?

Political Religious Other None

1. What is the setting of the mission? (Please check all that apply).

Hospital Clinic Urban Rural

1. How many days did the mission provide health care?

__________________Days

1. Has the mission visited this site previously? If yes, how many times?

__________________Times

1. How many missions does the organization send *to this site* per year?

__________________Missions

1. How many missions does the organization send internationally per year?

__________________Missions

1. How long has the mission organization been in existence?

__________________Years

1. What percentage of mission activities are directed toward health care?

0-15% 15-25% 25-50% 50-75% 75-100%

1. Please classify the patient population that you serve? Do you target any specific health concerns?

Adults Children Both

Specific concerns: ____________________________________

16) What is the native language of the population you serve?
